# Racial/ethnic differences in health-related quality of life among Hawaii adult population

**DOI:** 10.1186/s12955-020-01625-4

**Published:** 2020-12-09

**Authors:** Eunjung Lim, James Davis, Chathura Siriwardhana, Lovedhi Aggarwal, Allen Hixon, John J. Chen

**Affiliations:** 1 Department of Quantitative Health Sciences, John A. Burns School of Medicine, University of Hawaii, 651 Ilalo Street, Medical Education Building, Suite 411, Honolulu, HI 96813 USA; 2 Department of Family, Population & Preventive Medicine, Stony Brook University Medical Center, HSC L3, Rm 086, Stony Brook, NY 11794-8036 USA; 3 Department of Family Medicine, John A. Burns School of Medicine, University of Hawaii, Family Medicine at the Physician Center at Mililani, 95-390 Kuahelani AVE, Mililani, HI 96789 USA

**Keywords:** Race/ethnicity, Health-related quality of life, Andersen’s behavioral model, Self-rate general health, Mental health, Physical health

## Abstract

**Background:**

This study examined racial/ethnic differences in health-related quality of life (HRQOL) among adults and identified variables associated with HRQOL by race/ethnicity.

**Methods:**

This study was conducted under a cross-sectional design. We used the 2011–2016 Hawaii Behavioral Risk Factor Surveillance System data. HRQOL were assessed by four measures: self-rated general health, physically unhealthy days, mentally unhealthy days, and days with activity limitation. Distress was defined as fair/poor for general health and 14 days or more for each of the other three HRQOL measures. We conducted multivariable logistic regressions with variables guided by Anderson’s behavioral model on each distress measure by race/ethnicity.

**Results:**

Among Hawaii adults, 30.4% were White, 20.9% Japanese, 16.8% Filipino, 14.6% Native Hawaiian and Pacific Islander (NHPI), 5.9% Chinese, 5.2% Hispanics, and 6.2% Other. We found significant racial/ethnic differences in the HRQOL measures. Compared to Whites, Filipinos, Japanese, NHPIs, and Hispanics showed higher distress rates in general health, while Filipinos and Japanese showed lower distress rates in the other HRQOL measures. Although no variables were consistently associated with all four HRQOL measures across all racial/ethnic groups, history of diabetes were significantly associated with general health across all racial/ethnic groups and history of depression was associated with at least three of the HRQOL measure across all racial/ethnic groups.

**Conclusions:**

This study contributes to the literature on disparities in HRQOL and its association with other variables among diverse racial/ethnic subgroups. Knowing the common factors for HRQOL across different racial/ethnic groups and factors specific to different racial/ethnic groups will provide valuable information for identifying future public health priorities to improve quality of life and reduce health disparities.

## Background

Health-related quality of life (HRQOL) measures a person’s subjective perception of his/her life with a multi-dimensional concept encompassing physical and mental health, emotional well-being, and social functioning [1]. HRQOL has been used to predict the burden of chronic and preventable disease and disabilities [2]. Its importance has led to an increasing consensus for evaluating HRQOL in public health surveillance systems [3] and intervention studies [4].

A growing body of literature (or research) has revealed racial/ethnic differences in HRQOL. Most of the studies have focused on Blacks and Hispanics and described poor HRQOL among these racial groups compared to Whites [5–7]. However, the association with HRQOL has not been well documented with Native Hawaiians and other Pacific Islanders (NHPIs) or diverse Asian groups. Due to small sample sizes, these groups are often ignored or aggregated, which could mask their ethnic-based inequities. NHPI and Asian groups are the fastest-growing minorities in the U.S. [8] and are diverse in cultural tradition, socioeconomic status, English proficiency, and life experiences [9].

Numerous studies have been conducted to identify predictors of HRQOL and social and clinical determinants for HRQOL. For example, a study conducted among older adults using the National Health and Nutrition Examination Survey (NHANES) data found significant association between HRQOL and education, depression, and activities of daily living [10]. A study in Brazil showed significant differences in HRQOL by maternal education, social class, and household income among children [11]. However, very few studies have investigated the heterogeneity of demographic and social determinants of HRQOL across the NHPI and diverse Asian groups [12, 13].

To fill this gap, we explored important variables associated with HRQOL for these disaggregated and detailed racial/ethnic categories. Andersen’s behavioral model guided determination of candidate variables. The model has been used to delineate a comprehensive view of multiple influences from three groups of variables to HRQOL – predisposing, need, and enabling variables [10, 14]. *Predisposing variables* are social and cultural characteristics that exist prior to individuals’ disease including demographic and social structure (e.g., education). *Need variables* refer to conditions that laypeople or health care providers recognize as requiring medical treatment (e.g., chronic conditions). *Enabling variables* show logistical aspects of getting care which comprise medical personnel, health facilities, and patient’s personal means to get access to healthcare including financing factors and organizational factors (e.g., income).

We examined racial/ethnic differences in HRQOL among adults who live in Hawaii and identify important variables associated with HRQOL among six major racial/ethnic groups in Hawaii – White, Chinese, Filipino, Japanese, NHPI, and Hispanic. We hypothesized that race/ethnicity is significantly associated with HRQOL and the significance, magnitude and direction of variables guided by Anderson’s model on HRQOL differ by race/ethnicity.

## Methods

### Data source and study sample

We used the 2011–2016 data from the Hawaii Behavioral Risk Factor Surveillance System (BRFSS). The BRFSS is an annual cross-sectional ongoing state-based health survey for US non-institutionalized adults aged 18 years or older. The BRFSS collects data on sociodemographic variables, chronic illness, health behaviors, access to healthcare, and other health-related information in all 50 states in the US and the District of Columbia, Puerto Rico, and the US Virgin Islands. Further information about survey design and sampling method can be found on the BRFSS website (http://www.cdc.gov/BRFSS). For Hawaii BRFSS, the Hawaii state department of health (DOH) collects and distributes the BRFSS. We received detailed disaggregated race/ethnicity information from the Hawaii DOH and linked to the publicly available BRFSS data through the participants’ IDs. This study included 45,230 respondents after excluding 313 who did not provide their racial/ethnic information. The study received exempt certification from the University of Hawaii Internal Review Board (IRB).

### Variables

#### Health related quality of life measures

The HRQOL was assessed by the following four core questions developed by the Center for Disease Control and Prevention (http://www.cdc.gov/HRQOL/methods.htm): (1) General Health: “Would you say that in general your health is? (1 = Excellent, 2 = Very good, 3 = Good, 4 = Fair, 5 = Poor)”; (2) Physical Health: “For how many days during the past 30 days was your physical health not good?”; (3) Mental Health: “For how many days during the past 30 days was your mental health not good?”; and (4) Activity Limitation: “During the past 30 days, for about how many days did poor physical or mental health keep you from doing your usual activities, such as self-care, work, or recreation?” General health was further categorized as fair/poor (1) versus excellent/very good/good (0). We dichotomized the other HRQOL measures into ≥ 14 days or more (1) and < 14 days (0), as previously used in other studies [15]. For each of the HRQOL measures, distress status was defined as yes (1)/no (0).

#### Race/ethnicity

We used two variables to define race/ethnicity: primary race and Hispanic. If participants selected their primary race as any of NHPI, Chinese, Filipino, or Japanese, their race/ethnicity was defined as their primary race regardless of whether they identified themselves as Hispanic; otherwise, it was defined as Hispanic if they selected Hispanic, as non-Hispanic White if they selected White, and finally the race/ethnicity was assigned as Other. Therefore, we categorized race/ethnicity as White, Chinese, Filipino, Japanese, NHPI, Hispanic, and Other.

#### Variables guided by Anderson’s behavioral model

##### Predisposing variables

This study considered age, sex, ethnicity, marital status, and education as predisposing variables. Age was categorized into three groups: 18–44, 45–64, and ≥ 65 years. We categorized marital status into three groups as married/cohabitating, single, and separated/widowed/divorced. The categories for education were high school graduate or less, some college, and college graduate or more.

##### Enabling variables

Enabling variables include employment status, income, housing, residential area, and two healthcare access variables. Employment status was dichotomized into employed versus unemployed. Income was categorized to < $50,000 and ≥ $50,000. Since about 10% of participants (n = 4540) refused to report or did not know their income levels, we included a missing category for income. We defined housing status as owning vs other (renting or other arrangement). Residential area was categorized as Oahu (considered urban) vs other islands (considered rural). Two healthcare access variables were 1) having any health care coverage (yes vs. no) and 2) medical cost affordability defined by the question “Couldn’t see a doctor because of medical cost” (yes vs. no).

##### Need variables

The need variables in this study belong to two major groups—behavioral and clinical. Behavioral variables were exercise, heavy drinking, and smoking status (i.e., current smoking vs. former or never smoked). We defined exercise for a positive response on the following question: “During the past month, other than your regular job, did you participate in any physical activities or exercises such as running, calisthenics, golf, gardening, or walking for exercise?” We defined heavy drinking as having more than 14 drinks per week for men and more than 7 drinks per week for women (yes vs. no).

Clinical variables were obese/overweight status and history of eleven major chronic diseases (yes vs. no): heart attack, coronary heart diseases (CHD), stroke, asthma, cancer, chronic obstructive pulmonary disease (COPD), arthritis, depression, kidney disease, and diabetes—for simplicity, we excluded ‘history of’ in front of the chronic diseases in this manuscript. Obese/overweight was defined as body mass index greater than 25.0 kg/m^2^ (yes vs. no).

### Statistical analysis

All analyses were implemented in R version 3.6.1 using the *survey* package [16], incorporating the Hawaii BRFSS sampling design. We reported descriptive statistics by frequencies and weighted percentages; and assessed bivariate associations between variables and race/ethnicity using Rao-Scott’s chi-square tests. A logistic regression analysis was conducted to investigate racial/ethnic differences in distress for each of HRQOL measures. As subgroup analyses, multivariable logistic regression models were developed to identify important variables associated with HRQOL measures for each racial/ethnic group. We assessed variance inflation factor (VIF) to evaluate the multicollinearities of variables in the models; none had a VIF greater than 10. To adjust for the multiple testing issue using four HRQOL measures, *P* value less than 0.01 was considered statistically significant.

## Results

The study data comprised 30.4% White, 20.9% Japanese, 16.8% Filipino, 14.6% NHPI, 5.9% Chinese, 5.2% Hispanic, and 6.2% Other in Hawaii (Table 1). About 20% were aged 65 years or older; about half were males and half were married. The majority were employed, had insurance, and lived on Oahu. The distress rates among Hawaii adults were defined as 14.4% in general health, 9.4% in physical health, 8.6% in mental health, and 3.3% in activity limitation.

**Table 1:** Subject characteristics stratified by race/ethnicity

Variable	All n (weighted %)	Race/ethnicity, weighted column %						
		White n = 17,100 (30.4%)	Chinese n = 2222 (5.9%)	Filipino n = 5590 (16.8%)	Japanese n = 8800 (20.9%)	NHPI n = 6998 (14.6%)	Hispanic n = 2036 (5.2%)	Other n = 2484 (6.2%)
*Predisposing variable*								
Sex, male	21,848 (50.0)	54.0	49.8	46.7	46.1	47.7	54.6	54.1
Age								
18–44 years	13,975 (46.3)	43.6	40.1	52.1	27.9	63.4	63.9	57.8
45–64 years	17,334 (33.2)	35.2	34.7	32.7	37.9	25.9	26.7	29.7
≥ 65 years	13,921 (20.5)	21.2	25.2	15.2	34.2	10.7	9.4	12.5
Marital status								
Married	23,754 (54.5)	58.2	54.9	57.7	55.0	45.1	49.9	52.1
Single	9931 (27.6)	22.2	29.2	28.2	24.9	38.6	32.4	30.3
Separated/divorced/widowed	11,366 (17.8)	19.5	15.9	14.0	20.1	16.3	17.7	17.6
Education								
≤ High school graduate	14,245 (39.3)	31.9	27.5	47.9	30.3	59.4	49.9	37.0
Some college	12,574 (33.5)	34.2	31.4	34.6	34.7	28.3	34.0	36.7
College graduate	18,308 (27.3)	33.9	41.1	17.5	35.0	12.3	16.1	26.2
*Enabling variable*								
Employment, employed	25,208 (61.3)	62.8	57.2	68.1	54.4	61.5	62.7	62.0
Income								
< $50,000	21,107 (44.1)	38.5	35.5	55.0	34.2	57.9	2.4	44.1
≥ $50,000	19,723 (45.0)	52.5	51.2	32.2	53.5	32.3	38.4	43.6
Missing	4400 (10.9)	9.0	13.3	12.8	12.3	9.8	9.3	12.3
Housing status, own	26,350 (62.0)	62.3	79.0	57.0	78.8	46.8	44.5	50.9
Residential area, Oahu	22,726 (69.8)	59.3	92.0	71.3	78.9	65.8	62.2	80.3
Having insurance	41,826 (91.6)	92.3	93.4	90.8	95.5	87.1	88.1	89.3
Couldn’t see doctor because of medical cost	3874 (8.6)	8.2	4.9	11.2	2.9	13.5	13.5	10.3
*Need variable*								
Exercise	35,087 (79.2)	84.8	78.1	72.1	77.8	76.2	82.9	80.7
Heavy drinking	3378 (7.6)	10.1	3.5	4.8	5.1	10.6	10.3	6.6
Smoking, current	5760 (14.3)	14.1	6.3	12.4	10.4	22.9	18.5	18.0
H/O heart attack	1888 (3.2)	3.6	3.6	2.1	3.3	3.9	2.1	2.9
H/O CHD	1721 (3.0)	3.1	2.6	2.4	3.6	3.4	1.7	3.1
H/O stroke	1548 (2.8)	2.7	3.0	2.1	3.5	3.2	2.2	2.6
H/O asthma	7062 (16.2)	13.7	14.8	14.2	14.6	24.7	22.1	16.6
H/O cancer	6026 (9.1)	16.4	5.3	3.7	8.6	6.0	5.0	4.4
H/O COPD	2281 (4.1)	5.0	2.9	3.0	4.1	4.8	4.2	3.2
H/O arthritis	11,441 (20.1)	22.4	18.3	17.3	22.6	18.1	16.5	17.0
H/O depression	5795 (11.3)	16.1	7.0	6.8	7.0	12.3	17.7	11.3
H/O kidney disease	1832 (3.5)	3.5	2.9	2.9	3.8	4.3	3.5	3.1
H/O diabetes	4973 (9.8)	5.7	9.2	12.2	13.0	12.2	9.3	8.3
Obese/overweight	24,340 (56.6)	54.4	40.7	55.7	51.0	75.3	63.2	54.9
*HRQOL measure*								
General health: fair/poor	7103 (14.4)	11.2	12.8	13.9	14.9	21.0	16.9	13.1
Physical health^a^	4847 (9.4)	10.0	8.0	7.9	8.3	11.4	11.1	9.0
Mental health^a^	3889 (8.6)	9.1	8.0	5.9	5.9	12.0	14.0	10.7
Activity limitation^a^	1708 (3.3)	4.0	2.3	2.2	1.7	4.9	4.8	4.1

Race/ethnicity was significantly associated with all variables except kidney disease by Rao-Scott’s chi-square test at the significance level of 0.01

NHPI, Native Hawaiian/other Pacific Islander; H/O, history of; CHD, coronary heart disease; COPD, chronic obstructive pulmonary disease; HRQOL, health-related quality of life

^a^≥ 14 unhealthy days or more

All the variables in Table 1 revealed significant differences among racial/ethnic groups except for kidney disease. For example, more Hispanics and NHPIs were in the younger age group, while more Japanese were in the older age group. Regarding education, Chinese had the highest rate of college graduate while NHPI had the highest rate of high school or lower education level.

**Fig. 1:**
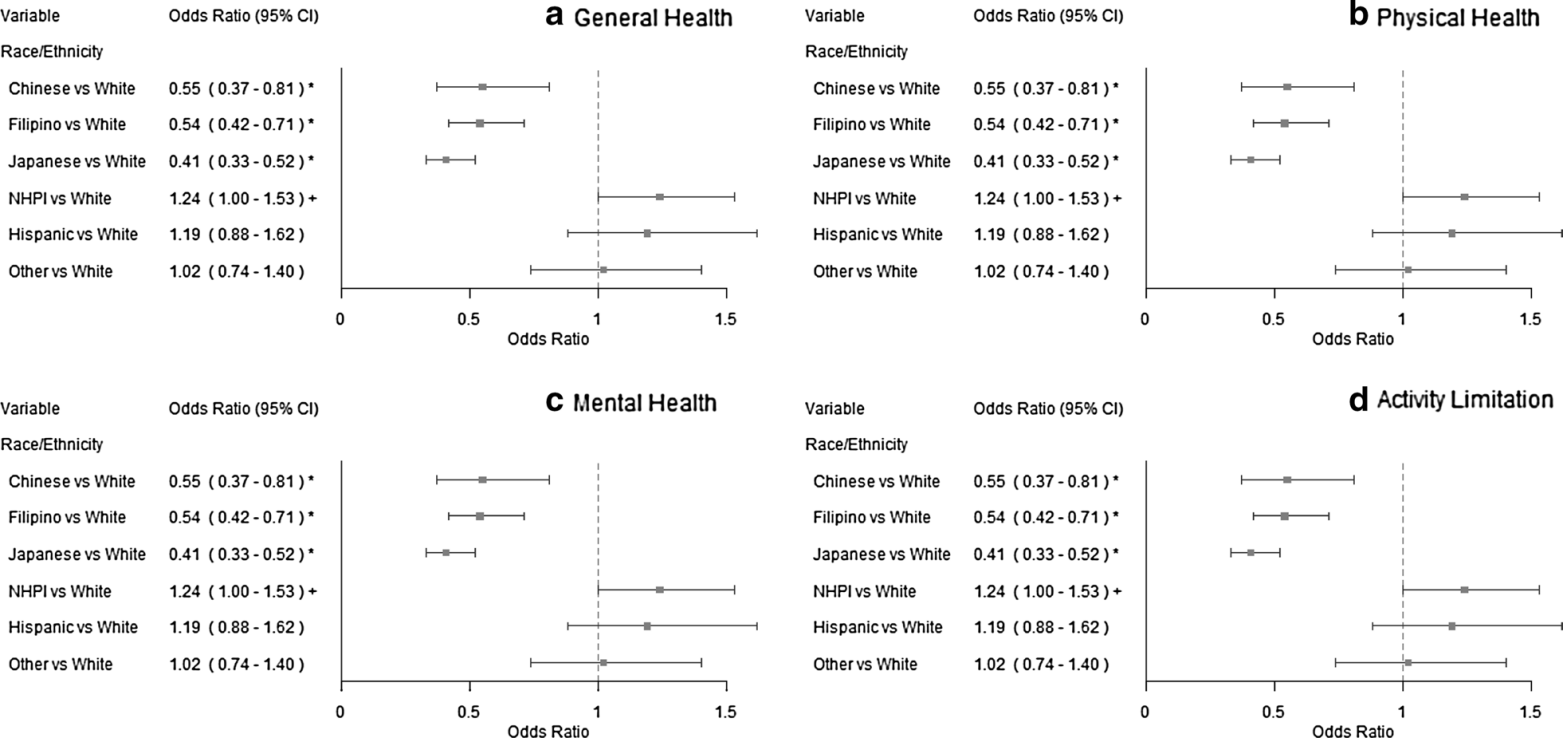
Odds ratio and 95% confidence interval (CI) for health-related quality of life measures. A logistic regression analysis was conducted on distress of each health-related quality of life measure with race/ethnicity, adjusting for the BRFSS complex sampling design. **a** General Health. Distress was defined as fair/poor in general health. **b** Physical Health. Distress was defined as having ≥ 14 physically unhealthy days in past 30 days. **c** Mental Health. Distress was defined as having ≥ 14 mentally unhealthy days in past 30 days. **d** Activity Limitation. Distress was defined as ≥ 14 days with activity limitation in past 30 days. NHPI = Native Hawaiian/other Pacific Islander. ^+^*P* value < 0.05. *P value < 0.01

The distress rates varied across race/ethnicity (Table 1). Distress rates ranged from 11.2% (White) to 21.0% (NHPI) for general health; 7.9% (Filipino) to 11.4% (NHPI) for physical health; 5.9% (Filipino and Japanese) to 14.0% (Hispanic) for mental health; and 1.7% (Japanese) to 4.9% (NHPI) for activity limitation. Logistic regressions on distress also confirmed these findings (Fig. 1). For general health, the odds of reporting fair/poor general health in NHPI, Hispanic, Japanese, and Filipino were 2.10, 1.61, 1.39, and 1.28 times greater than White. Compared to Whites, the odds of being distressed in physical health were lower among Filipinos (odds ratio [OR] = 0.77) and Japanese (OR = 0.82) but higher among NHPIs (OR = 1.15). For mental health, NHPIs and Hispanics had increased odds of being distressed (ORs = 1.37 and 1.63, respectively), but Filipino and Japanese had decreased odds (both ORs = 0.63), compared to White. The odds of having ≥ 14 unhealthy days in activity limitation were lower in Chinese (OR = 0.55), Filipino (OR = 0.54), and Japanese (OR = 0.41) relative to White.

### Subgroup analysis by race/ethnicity

#### General health

The relative importance of variables for general health varies by race/ethnicity (Table 2). Diabetes was the only common variable associated with general health across all racial/ethnic groups. Not all but across five racial/ethnic groups, associated with general health were three other variables: exercise, depression, arthritis, and kidney disease. Exercise was negatively associated with distress in general health except for Filipino, but depression, kidney disease, and arthritis were positively associated except for Hispanic.

**Table 2:** Adjusted odds ratios (and 95% confidence intervals) from multivariable logistic regression for general health by race/ethnicity

Variable	White	Chinese	Filipino	Japanese	NHPI	Hispanic
*Predisposing variable*						
Sex, male	1.22 (1.02–1.45)^+^	1.38 (0.92–2.06)	0.80 (0.62–1.05)	1.08 (0.88–1.32)	1.12 (0.92–1.37)	1.29 (0.84–1.97)
Age (Ref: 18–44 years)						
45–64 years	1.48 (1.13–1.93)*	1.36 (0.92–2.06)	1.38 (0.97–1.96)	1.81 (1.30–2.51)*	1.29 (0.99–1.67)	2.78 (1.70–4.56)*
≥ 65 years	1.21 (0.87–1.68)	1.38 (0.67–2.85)	1.03 (0.65–1.62)	1.45 (0.99–2.10)	1.16 (0.81–1.66)	2.70 (1.38–5.31)*
Marital status (Ref: married)						
Single	1.07 (0.82–1.41)	1.07 (0.60–1.91)	1.19 (0.80–1.77)	0.96 (0.72–1.26)	0.95 (0.73–1.23)	1.22 (0.70–2.14)
Separated/divorced/widowed	1.02 (0.83–1.24)	0.88 (0.53–1.47)	1.31 (0.93–1.84)	0.97 (0.77–1.22)	0.89 (0.68–1.17)	0.91 (0.53–1.55)
Education (Ref: ≤ high school)						
Some college	0.74 (0.60–0.91)*	0.85 (0.53–1.35)	0.91 (0.68–1.22)	0.82 (0.66–1.03)	0.89 (0.72–1.11)	1.07 (0.67–1.71)
College graduate	0.51 (0.41–0.57)*	0.59 (0.36–0.97)^+^	0.92 (0.66–1.29)	0.61 (0.49–0.77)*	0.73 (0.54–0.98)^+^	0.57 (0.33–0.99)^+^
*Enabling variable*						
Employment (Ref: unemployed)						
Employed	0.47 (0.39–0.57)*	0.72 (0.45–1.20)	0.65 (0.47–0.91)^+^	0.77 (0.62–0.97)^+^	0.93 (0.75–1.16)	0.63 (0.40–0.98)^+^
Income (Ref: < $50,000)						
≥ $50,000	0.60 (0.48–0.73)*	0.88 (0.55–1.41)	0.74 (0.54–1.01)	0.69 (0.55–0.86)*	0.84 (0.66–1.08)	0.59 (0.32–1.07)
Missing	0.92 (0.63–1.33)	1.17 (0.58–2.38)	0.84 (0.52–1.36)	1.54 (1.16–2.06)*	0.88 (0.60–1.27)	0.66 (0.26–1.68)
Housing status (Ref: own)						
Other	1.13 (0.89–1.43)	1.43 (0.85–2.42)	0.89 (0.66–1.21)	1.30 (1.02–1.65)^+^	1.08 (0.84–1.39)	1.24 (0.71–2.15)
Residential area, Oahu	0.99 (0.83–1.17)	0.73 (0.45–1.20)	1.22 (0.94–1.57)	0.98 (0.81–1.18)	1.18 (0.97–1.43)	1.98 (1.29–3.04)*
Have healthcare coverage	1.06 (0.74–1.52)	1.10 (0.48–2.23)	0.68 (0.44–1.04)	1.20 (0.74–1.96)	1.14 (0.80–1.64)	0.59 (0.30–1.19)
Couldn’t see doctor because of medical cost	1.62 (1.23–2.13)*	1.04 (0.48–2.23)	1.19 (0.80–1.77)	2.43 (1.53–3.86)*	1.40 (1.03–1.91)^+^	1.83 (1.04–3.20)^+^
*Need variable*						
Exercise	0.38 (0.31–0.46)*	0.51 (0.34–0.77)*	0.69 (0.52–0.93)^+^	0.51 (0.42–0.62)*	0.49 (0.40–0.62)*	0.45 (0.29–0.71)*
Heavy drinking	0.73 (0.54–0.98)^+^	1.38 (0.46–4.18)	0.67 (0.38–1.20)	1.22 (0.81–1.84)	1.12 (0.80–1.56)	1.00 (0.51–1.95)
Smoking (Ref: current)						
Former/never	0.85 (0.67–1.09)	0.64 (0.31–1.33)	0.55 (0.37–0.83)*	1.24 (0.90–1.70)	0.61 (0.48–0.78)*	0.62 (0.34–1.14)
H/O heart attack	1.85 (1.30–2.62)*	1.28 (0.46–3.53)	1.81 (0.95–3.46)	1.67 (1.09–2.57)^+^	1.61 (1.00–2.60)	1.55 (0.54–4.47)
H/O CHD	1.57 (1.10–2.25)^+^	2.13 (0.86–5.28)	2.08 (1.21–3.59)*	2.01 (1.35–3.00)*	1.31 (0.78–2.19)	2.87 (1.00–8.26)^+^
H/O stroke	2.49 (1.59–3.89)*	1.65 (0.38–7.20)	0.93 (0.44–1.98)	1.63 (1.11–2.41)^+^	1.79 (1.10–2.93)^+^	1.37 (0.50–3.78)
H/O asthma	1.31 (1.05–1.64)^+^	1.58 (0.99–2.52)	1.19 (0.85–1.65)	1.13 (0.86–1.48)	1.11 (0.88–1.40)	1.36 (0.79–2.34)
H/O cancer	1.38 (1.13–1.69)*	2.56 (1.35–4.86)*	2.23 (1.34–3.70)*	1.55 (1.20–2.01)*	1.43 (1.03–2.00)^+^	1.18 (0.56–2.47)
H/O COPD	2.20 (1.70–2.84)*	3.86 (1.69–8.84)*	1.87 (1.09–3.21)^+^	1.87 (1.34–2.63)*	2.80 (1.92–4.07)*	1.25 (0.65–2.43)
H/O arthritis	1.86 (1.55–2.22)*	2.06 (1.32–3.23)*	1.76 (1.31–2.38)*	1.94 (1.59–2.36)*	1.64 (1.30–2.07)*	1.68 (1.08–2.60)^+^
H/O depression	2.58 (2.12–3.13)*	2.64 (1.38–5.06)*	1.87 (1.18–2.97)*	2.16 (1.64–2.84)*	2.14 (1.63–2.82)*	1.76 (1.05–2.97)^+^
H/O kidney disease	2.04 (1.38–3.02)*	3.02 (1.44–6.31)*	3.96 (2.16–7.26)*	2.50 (1.77–3.53)*	2.67 (1.76–4.03)*	2.02 (0.74–5.49)
H/O diabetes	2.31 (1.79–2.98)*	1.96 (1.20–3.21)*	2.44 (1.72–3.45)*	2.64 (2.11–3.31)*	2.27 (1.76–2.92)*	2.85 (1.67–4.87)*
Obese/overweight	1.48 (1.23–1.77)*	1.21 (0.81–1.81)	1.23 (0.94–1.60)	1.43 (1.18–1.74)*	1.45 (1.13–1.85)*	1.48 (0.88–2.48)

General health was dichotomized as fair/poor (1) vs. excellent/very good/good (0). A multivariable logistic regression with quasi-binomial distribution was conducted, accounting for the complex sampling design

NHPI, Native Hawaiian/other Pacific Islander; H/O, history of; CHD, coronary heart disease; COPD, chronic obstructive pulmonary disease; Ref, reference

^+^*P* value < 0.05; *P value < 0.01

Predictors specific to White were two from predisposing variables (age and education), three from enabling variables (employment, income, and medical cost affordability) and six from need variables (obese/overweight, heart attack, stroke, cancer, COPD, and kidney disease). Those aged 45–64 years had 1.48 times higher odds of reporting fair/poor general health compared to those aged 18–44 years. Higher education level was negatively associated with distress in general health. People with at least some college level education were less likely to report fair/poor health compared to those with high school graduate or less education. Employed people or those with higher income were less likely to be distressed compared to their counterparts. We also explored bivariate association between education and employment status. Higher education was significantly associated with higher employment rate (results are not shown). Thus, these variables might independently affect general health. Those who couldn’t see a doctor due to medical cost were 1.62 times more likely to be distressed than those who can afford.

Specific to Chinese, three need variables were significant: cancer, COPD, and kidney disease. Those with these diseases were more likely to be distressed than those without it. Across the racial/ethnic groups, the effect of COPD was the highest among Chinese (OR = 3.86).

Specific to Filipino, four need variables were significant: smoking, CHD, cancer, and kidney disease. Smokers are more likely to report distress in general health than non-smokers. CHD, cancer, and kidney disease were risk factors for distress in general health; the effect of kidney disease was the highest for Filipino among the racial/ethnic groups (OR = 3.96).

Predictors specific to Japanese were two from predisposing variables (age, education), two from enabling variables (income and medical cost affordability), and four from need variables (obese/overweight, CHD, cancer, COPD, and kidney disease). Those aged 45–64 years have 1.81 times higher odds of reporting fair/poor general health compared to those aged 18–44 years. People with college graduate level education were less likely to report distress in general health compared to those with high school graduate or less education. Those having ≥ $50,000 were less likely to be distressed compared to those having < $50,000. Different from the other racial/ethnic groups, Japanese who refused to disclose their income were 1.54 times more likely to be distressed in general health than those having < $50,000. Those who couldn’t see a doctor due to medical cost were 2.43 times more likely to be distressed than those who can. Obese or overweight people had a 1.43 times higher distress rate than people having normal or underweight.

Specific to NHPI, four need variables were significant: smoking, obese/overweight COPD, and kidney disease. Smokers are more likely to report distress in general health than non-smokers. The odds of being distressed in general health among obese or overweight people were 1.45 times higher than people having normal or underweight.

Predictors specific to Hispanic were age (predisposing), residential area (enabling), and heavy drinking (need). Those aged 45–64 years and 65 years or older have 2.78 and 2.70 times higher odds of reporting fair/poor general health compared to those aged 18–44 years, respectively. Oahu residents are 1.98 times more likely to be distressed in general health compared to people who live in the other islands.

#### Physical health

The relative importance of variables for physical health differs by race/ethnicity (Table 3). None was significantly associated with physical health across all the racial/ethnic groups.

**Table 3:** Adjusted odds ratios (and 95% confidence intervals) from multivariable logistic regression for physical health by race/ethnicity

Variable	White	Chinese	Filipino	Japanese	NHPI	Hispanic
*Predisposing variable*						
Sex, Male	1.13 (0.94–1.35)	0.82 (0.48–1.39)	0.83 (0.59–1.16)	0.85 (0.68–1.07)	1.67 (1.27–2.19)*	1.50 (0.95–2.38)
Age (Ref: 18–44 years)						
45–64 years	1.27 (0.98–1.63)	1.13 (0.47–2.69)	1.96 (1.27–3.02)*	2.01 (1.32–3.06)*	1.13 (0.78–1.62)	1.49 (0.85–2.59)
≥ 65 years	0.99 (0.73–1.35)	0.68 (0.24–1.92)	1.92 (1.08–3.44)^+^	1.25 (0.78–2.02)	0.72 (0.42–1.24)	2.06 (0.97–4.40)
Marital status (Ref: married)						
Single	1.35 (1.03–1.79)^+^	0.77 (0.34–1.75)	1.26 (0.77–2.08)	0.84 (0.59–1.19)	0.57 (0.40–0.81)*	1.57 (0.79–3.15)
Separated/divorced/widowed	1.07 (0.87–1.31)	1.34 (0.74–2.43)	1.01 (0.67–1.52)	1.02 (0.77–1.34)	0.89 (0.64–1.24)	1.29 (0.70–2.37)
Education (Ref: ≤ high school)						
Some college	1.04 (0.83–1.31)	0.61 (0.32–1.17)	1.05 (0.74–1.49)	0.96 (0.73–1.27)	0.86 (0.64–1.15)	0.86 (0.51–1.46)
College graduate	0.84 (0.67–1.06)	0.55 (0.31–0.98)^+^	0.76 (0.52–1.11)	0.92 (0.69–1.23)	0.68 (0.46–1.00)	0.51 (0.26–1.01)
*Enabling variable*						
Employment (Ref: unemployed)						
Employed	0.57 (0.47–0.69)*	0.44 (0.24–0.82)*	0.75 (0.50–1.11)	0.54 (0.40–0.72)*	0.64 (0.48–0.86)*	0.60 (0.35–1.03)
Income (Ref: < $50,000)						
≥ $50,000	0.78 (0.63–0.97)^+^	1.23 (0.72–2.12)	0.65 (0.44–0.94)^+^	0.71 (0.54–0.94)^+^	0.81 (0.57–1.15)	1.09 (0.58–2.07)
Missing	0.87 (0.61–1.24)	0.78 (0.28–2.15)	1.15 (0.63–2.08)	0.86 (0.57–1.29)	0.88 (0.52–1.47)	0.94 (0.36–2.42)
Housing status (Ref: own)						
Other	1.05 (0.83–1.33)	0.75 (0.38–1.46)	1.11 (0.77–1.60)	1.51 (1.15–1.99)*	1.09 (0.80–1.49)	0.69 (0.39–1.24)
Residential area, Oahu	0.90 (0.76–1.07)	1.67 (0.88–3.13)	0.96 (0.70–1.31)	0.93 (0.74–1.17)	0.91 (0.70–1.18)	0.78 (0.47–1.28)
Have healthcare coverage	1.53 (1.06–2.20)^+^	0.81 (0.16–4.03)	1.43 (0.69–2.93)	1.69 (0.88–3.21)	1.48 (0.87–2.53)	1.29 (0.61–2.74)
Couldn’t see doctor because of medical cost	1.75 (1.32–2.32)*	1.34 (0.47–3.78)	1.50 (0.93–2.44)	1.42 (0.86–2.34)	1.21 (0.85–1.73)	1.76 (0.97–3.19)
*Need variable*						
Exercise	0.34 (0.28–0.41)*	0.39 (0.23–0.66)*	0.93 (0.68–1.29)	0.55 (0.43–0.70)*	0.43 (0.33–0.57)*	0.79 (0.46–1.36)
Heavy drinking	0.75 (0.56–1.00)	0.32 (0.05–1.89)	0.82 (0.44–1.54)	1.17 (0.64–2.14)	0.90 (0.57–1.43)	1.46 (0.71–3.00)
Smoking (Ref: current)						
Former/never	0.86 (0.67–1.10)	1.26 (0.56–2.87)	1.03 (0.60–1.75)	1.19 (0.83–1.71)	0.92 (0.68–1.26)	0.90 (0.51–1.61)
H/O heart attack	1.29 (0.96–1.72)	0.31 (0.06–1.63)	1.15 (0.57–2.32)	0.79 (0.46–1.36)	1.30 (0.79–2.14)	1.01 (0.37–2.74)
H/O CHD	1.31 (0.96–1.80)	0.97 (0.21–4.36)	1.29 (0.67–2.48)	1.83 (1.12–3.00)^+^	1.05 (0.59–1.89)	0.97 (0.34–2.79)
H/O stroke	1.97 (1.39–2.78)*	4.37 (1.12–17.08)^+^	2.52 (1.36–4.69)*	1.06 (0.66–1.69)	1.71 (1.01–2.89)^+^	2.22 (0.79–6.28)
H/O asthma	1.08 (0.87–1.34)	2.77 (1.59–4.85)*	0.82 (0.54–1.27)	1.27 (0.91–1.76)	1.44 (1.08–1.91)^+^	1.50 (0.86–2.61)
H/O cancer	1.37 (1.12–1.66)*	1.62 (0.83–3.16)	1.85 (1.02–3.38)^+^	1.74 (1.29–2.34)*	1.71 (1.13–2.58)^+^	0.81 (0.36–1.83)
H/O COPD	2.27 (1.66–3.11)*	3.72 (1.64–8.44)*	1.54 (0.80–2.96)	1.97 (1.32–2.94)*	3.09 (1.93–4.97)*	2.83 (1.27–6.29)^+^
H/O arthritis	2.25 (1.88–2.68)*	2.03 (1.22–3.38)*	2.69 (1.91–3.79)*	2.53 (2.03–3.16)*	1.89 (1.40–2.55)*	1.45 (0.83–2.53)
H/O depression	2.23 (1.84–2.70)*	1.45 (0.69–3.08)	3.49 (2.24–5.45)*	2.33 (1.68–3.23)*	2.05 (1.48–2.83)*	2.68 (1.51–4.73)*
H/O kidney disease	1.24 (0.90–1.71)	3.98 (1.87–8.46)*	3.14 (1.80–5.46)*	1.54 (0.97–2.47)	1.71 (1.02–2.88)^+^	2.56 (0.94–6.93)
H/O diabetes	1.28 (0.99–1.66)	1.34 (0.73–2.47)	1.60 (1.09–2.34)^+^	1.44 (1.09–1.89)*	1.45 (1.07–1.97)^+^	2.13 (1.09–4.18)^+^
Obese/overweight	1.14 (0.95–1.36)	1.07 (0.62–1.82)	1.20 (0.86–1.67)	1.09 (0.86–1.37)	1.31 (0.96–1.80)	0.96 (0.60–1.54)

Physical health was dichotomized as ≥ 14 unhealthy days (1) vs. < 14 unhealthy days (0). A multivariable logistic regression with quasi-binomial distribution was conducted, accounting for the complex sampling design

NHPI, Native Hawaiian/other Pacific Islander; H/O, history of; CHD, coronary heart disease; COPD, chronic obstructive pulmonary disease; Ref, reference

^+^*P* value < 0.05; *P value < 0.01

Predictors specific to White were one from predisposing variables, one from enabling variables, and five from need variables: employment (OR = 0.57), medical cost affordability (OR = 1.75), exercise (OR = 0.34), stroke (OR = 1.97), cancer (OR = 1.37), COPD (OR = 2.27), arthritis (OR = 2.25), and depression (OR = 2.23). Medical cost affordability was associated with physical health only for White and those who couldn’t see a doctor due to medical cost were 1.75 times more likely to be distressed in physical health than those who can afford.

Specific to Chinese, one from enabling variables and five from need variables were significantly associated with physical health: employment (OR = 0.44), exercise (OR = 0.39), asthma (OR = 2.77), COPD (OR = 3.72), arthritis (OR = 2.03), and kidney disease (OR = 3.98). The effects of COPD and kidney disease were the highest among all racial/ethnic groups, and asthma was significant only for Chinese. In addition, the effect of stroke, though not significant at 0.01 (P = 0.034), but was the highest for Chinese among the racial/ethnic groups (OR = 4.37).

Specific to Filipino, one from predisposing variables and four from need variables were significant: age, stroke (OR = 2.52), arthritis (OR = 2.69), depression (OR = 3.49), and kidney disease (OR = 3.14). People aged 45–64 years had 1.96 times higher odds of distress compared to those aged 18–44 years. The effects of arthritis and depression were the highest among the racial/ethnic groups.

Predictors specific to Japanese were one from predisposing variables, two from enabling variables, and five from need variables: age, employment (OR = 0.54), housing status, exercise (OR = 0.55), cancer (OR = 1.74), COPD (OR = 1.97), arthritis (OR = 2.53), depression (OR = 2.33), and diabetes (OR = 1.44). Those aged 45–64 years have 2.01 times higher odds of distress in physical health compared to those aged 18–44 years. Housing status and diabetes were significant only for Japanese. Those who rent or live in other arrangements were 1.51 times more likely to be distressed than those who own their home.

Predictors specific to NHPI were two from predisposing variables, one from enabling variables, and four from need variables: sex (OR = 1.67), marital status (OR = 0.57), employment (OR = 0.64), exercise (OR = 0.43), COPD (OR = 3.09), arthritis (OR = 1.89), and depression (OR = 2.05). Sex and marital status were significantly associated with physical health only for Japanese. Men were 1.67 times more likely to report distress in physical health than women, and single people were less likely to be distressed in physical health than married people.

Specific to Hispanic, only one need variable, depression, appeared to be significant. People with depression were 2.68 times more likely to report distress in physical health than people without it.

#### Mental health

Again, race/ethnicity plays an important role for mental health (Table 4). Depression was the only common variable significantly associated with mental health across all the racial/ethnic groups, with ORs of 4.50 to 9.81 of distress in mental health among people with depression relative to those without.

**Table 4:** Adjusted odds ratios (and 95% confidence intervals) from multivariable logistic regression for mental health by race/ethnicity

Variable	White	Chinese	Filipino	Japanese	NHPI	Hispanic
*Predisposing variable*						
Sex, Male	0.91 (0.74–1.12)	0.73 (0.43–1.24)	0.99 (0.68–1.45)	1.06 (0.80–1.40)	1.26 (0.95–1.65)	0.72 (0.42–1.23)
Age (Ref: 18–44 years)						
45–64 years	0.76 (0.59–0.98)^+^	0.82 (0.46–1.47)	0.59 (0.38–0.91)^+^	1.17 (0.81–1.69)	0.76 (0.55–1.06)	0.74 (0.43–1.27)
≥ 65 years	0.33 (0.24–0.46)*	0.37 (0.15–0.88)^+^	0.73 (0.40–1.32)	0.62 (0.37–1.02)	0.34 (0.19–0.60)*	0.56 (0.24–1.29)
Marital Status (Ref: married)						
Single	1.04 (0.80–1.36)	1.24 (0.65–2.36)	1.38 (0.86–2.21)	1.15 (0.80–1.67)	1.01 (0.73–1.41)	0.87 (0.44–1.74)
Separated/divorced/widowed	1.08 (0.84–1.39)	1.16 (0.58–2.31)	1.35 (0.78–2.33)	1.14 (0.81–1.60)	1.18 (0.86–1.64)	0.65 (0.35–1.21)
Education (Ref: ≤ high school)						
Some college	0.99 (0.78–1.26)	0.91 (0.45–1.85)	0.83 (0.56–1.24)	1.01 (0.71–1.43)	1.25 (0.95–1.66)	0.63 (0.35–1.15)
College graduate	0.67 (0.52–0.86)*	0.90 (0.45–1.80)	1.13 (0.71–1.79)	1.26 (0.89–1.77)	0.92 (0.63–1.35)	0.59 (0.29–1.20)
*Enabling variable*						
Employment (Ref: unemployed)						
Employed	0.79 (0.64–0.97)^+^	0.57 (0.33–0.97)^+^	0.74 (0.50–1.08)	0.93 (0.70–1.24)	0.75 (0.57–0.99)^+^	0.89 (0.51–1.55)
Income (Ref: < $50,000)						
≥ $50,000	0.80 (0.62–1.02)	1.04 (0.55–1.95)	1.54 (0.98–2.41)	0.69 (0.50–0.96)^+^	0.80 (0.57–1.12)	0.77 (0.34–1.72)
Missing	1.07 (0.69–1.66)	0.92 (0.33–2.56)	0.74 (0.36–1.51)	0.91 (0.55–1.50)	0.80 (0.49–1.31)	0.28 (0.10–0.76)^+^
Housing status (Ref: own)						
Other	0.86 (0.67–1.10)	0.88 (0.47–1.63)	1.23 (0.8–1.88)	1.12 (0.79–1.60)	1.07 (0.77–1.48)	1.00 (0.52–1.92)
Residential area, Oahu	1.14 (0.94–1.39)	1.54 (0.82–2.91)	1.02 (0.72–1.46)	0.92 (0.70–1.20)	1.09 (0.85–1.40)	1.16 (0.73–1.85)
Have healthcare coverage	0.90 (0.61–1.30)	0.43 (0.22–0.85)^+^	1.08 (0.60–1.93)	1.07 (0.55–2.09)	1.29 (0.89–1.88)	0.88 (0.42–1.84)
Couldn’t see doctor because of medical cost	2.14 (1.61–2.85)*	4.36 (1.99–9.53)*	1.72 (0.98–3.04)	2.59 (1.51–4.46)*	1.97 (1.43–2.72)*	1.41 (0.81–2.47)
*Need variable*						
Exercise	0.56 (0.44–0.72)*	1.06 (0.59–1.90)	0.78 (0.51–1.20)	0.70 (0.53–0.93)^+^	0.72 (0.55–0.95)^+^	0.66 (0.40–1.09)
Heavy drinking	1.08 (0.81–1.42)	1.81 (0.59–5.52)	1.60 (0.78–3.29)	1.91 (1.22–2.99)*	1.43 (0.98–2.08)	1.09 (0.52–2.30)
Smoking (Ref: current)						
Former/never	0.71 (0.55–0.91)*	0.44 (0.23–0.83)^+^	0.55 (0.34–0.89)^+^	0.65 (0.44–0.98)^+^	0.73 (0.56–0.96)^+^	0.63 (0.31–1.26)
H/O heart attack	1.68 (1.18–2.38)*	1.06 (0.19–5.81)	2.24 (0.89–5.66)	1.31 (0.69–2.47)	1.36 (0.76–2.44)	4.29 (1.34–13.72)^+^
H/O CHD	1.35 (0.88–2.07)	1.80 (0.28–11.58)	1.07 (0.50–2.26)	1.46 (0.81–2.66)	0.95 (0.53–1.72)	0.25 (0.06–1.00)^+^
H/O stroke	1.12 (0.71–1.77)	0.49 (0.10–2.48)	1.06 (0.34–3.26)	1.11 (0.65–1.91)	1.63 (0.84–3.18)	1.13 (0.32–4.00)
H/O asthma	1.16 (0.90–1.49)	1.33 (0.69–2.57)	0.97 (0.61–1.56)	1.20 (0.81–1.78)	1.37 (1.03–1.82)^+^	2.53 (1.41–4.53)*
H/O cancer	0.99 (0.78–1.25)	0.46 (0.22–0.97)^+^	1.86 (0.87–3.97)	1.57 (0.98–2.54)	1.04 (0.66–1.64)	1.03 (0.53–2.00)
H/O COPD	1.38 (1.01–1.89)^+^	1.46 (0.60–3.57)	0.50 (0.19–1.29)	1.66 (1.00–2.76)	2.07 (1.19–3.59)*	1.18 (0.54–2.56)
H/O arthritis	1.31 (1.07–1.61)^+^	1.37 (0.70–2.68)	1.26 (0.83–1.91)	1.58 (1.18–2.10)*	1.69 (1.22–2.36)*	1.17 (0.64–2.11)
H/O depression	5.94 (4.86–7.26)*	8.34 (4.77–14.61)*	6.88 (4.46–10.6)*	4.77 (3.51–6.48)*	4.50 (3.41–5.94)*	9.81 (6.02–15.99)*
H/O kidney disease	1.52 (0.99–2.31)	0.89 (0.30–2.71)	1.81 (0.80–4.08)	1.67 (1.02–2.74)^+^	1.16 (0.65–2.07)	0.51 (0.22–1.18)
H/O diabetes	1.02 (0.75–1.38)	0.91 (0.37–2.26)	1.09 (0.65–1.82)	0.81 (0.56–1.18)	1.03 (0.73–1.45)	0.88 (0.44–1.75)
Obese/overweight	1.24 (1.01–1.52)^+^	0.89 (0.54–1.47)	1.17 (0.82–1.68)	0.85 (0.65–1.11)	0.96 (0.71–1.30)	0.63 (0.37–1.07)

Mental health was dichotomized as ≥ 14 unhealthy days (1) vs. < 14 unhealthy days (0). A multivariable logistic regression with quasi-binomial distribution was conducted, accounting for the complex sampling design

NHPI, Native Hawaiian/other Pacific Islander; H/O, History of; CHD, Coronary heart disease; COPD, chronic obstructive pulmonary disease; Ref, reference

^+^*P* value < 0.05; *P value < 0.01

Specific to White, two from predisposing variables, one from enabling variables, and three from need variables were significant: age, education, medical cost affordability, exercise, smoking, and heart attack. People aged 65 years or older are less likely to be distressed compared to those aged 18–44 years (OR = 0.33). People with at least some college level education were less likely to be distressed in mental health compared to those with high school graduate or less education (OR = 0.67). Those who couldn’t see a doctor due to medical cost were 2.14 times more likely to be mentally distressed than those who can. Those who exercise were less likely to report distress in mental health than those who do not (OR = 0.56). The odds of distress among people who do not smoke are lower by a factor of 0.71 than current smokers. Education, exercise, and smoking were significantly associated with distress in mental health only for White.

Specific to Chinese, beside depression, only one enabling variable, medical cost affordability, was significant. People who couldn’t see a doctor due to medical cost were 4.36 times more likely to be mentally distressed than those who can. Smoking did not meet the significance level at 0.01 (*P* = 0.012) but the odds of distress in mental health among non-smokers was the smallest among all racial/ethnic groups compared to smokers (OR = 0.44). Similarly, having health coverage did not meet the significance level at 0.01 (P = 0.016) but the OR was the smallest among the racial/ethnic groups (OR = 0.43).

Besides depression, the common variable, none of the other variables appeared to be significant, specific to Filipinos. People with depression was 6.88 times more likely to be distressed than those without it.

Predictors specific to Japanese were one from enabling variables and two from need variables: medical cost affordability, heavy drinking and arthritis (OR = 1.58). Those who couldn’t see a doctor due to medical cost were 2.59 times more likely to be distressed than those who can. Heavy drinking was positively associated with mental health only for Japanese (OR = 1.91).

Specific to NHPI, one from predisposing variables, one from enabling variables, and two from need variables were significant: age, medical cost affordability, COPD (OR = 2.07) and arthritis (OR = 1.69). People aged 65 years or older are less likely to be distressed, compared to those aged 18–44 years (OR = 0.34). Those who couldn’t see a doctor due to medical cost were 1.97 times more likely to be distressed than those who can.

A predictor specific to Hispanic was a need variable, asthma. It was associated with mental health only for Hispanic, and people with asthma was 2.53 times more likely to be mentally distressed than those without.

#### Activity limitation

Race/ethnicity affects the importance and significance of variables (Table 5). Depression was the only common variable associated with activity limitation across all the racial/ethnic groups, with ORs of 5.23 to 18.04 of distress in activity limitation.

**Table 5:** Adjusted odds ratios (and 95% confidence intervals) from multivariable logistic regression for activity limitation by race/ethnicity

Variable	White	Chinese	Filipino	Japanese	NHPI	Hispanic
*Predisposing variable*						
Sex, male	1.09 (0.81–1.45)	0.79 (0.32–1.93)	1.95 (1.09–3.48)^+^	1.17 (0.71–1.92)	1.89 (1.14–3.14)^+^	1.71 (0.86–3.40)
Age (Ref: 18–44 years)						
45–64 years	1.22 (0.84–1.78)	1.36 (0.45–4.15)	0.56 (0.29–1.09)	1.37 (0.64–2.92)	0.82 (0.48–1.42)	0.58 (0.23–1.47)
≥ 65 years	0.44 (0.27–0.72)*	0.35 (0.07–1.62)	0.34 (0.15–0.80)^+^	0.61 (0.25–1.49)	0.20 (0.08–0.50)*	0.24 (0.07–0.87)^+^
Marital status (Ref: married)						
Single	1.18 (0.77–1.80)	0.68 (0.21–2.21)	0.62 (0.29–1.34)	0.99 (0.55–1.78)	0.60 (0.36–1.02)	1.53 (0.51–4.62)
Separated/divorced/widowed	0.84 (0.62–1.15)	1.25 (0.41–3.79)	0.77 (0.36–1.63)	1.20 (0.67–2.14)	0.78 (0.48–1.27)	1.04 (0.35–3.06)
Education (Ref: ≤ high school)						
Some college	1.05 (0.76–1.45)	0.25 (0.07–0.87)^+^	0.49 (0.26–0.92)^+^	0.75 (0.40–1.38)	0.80 (0.53–1.22)	0.66 (0.23–1.87)
College graduate	0.85 (0.61–1.19)	0.48 (0.17–1.40)	1.00 (0.50–2.00)	1.33 (0.74–2.39)	0.47 (0.23–0.93)^+^	0.54 (0.17–1.72)
*Enabling variable*						
Employment (Ref: unemployed)						
Employed	0.30 (0.21–0.41)*	0.32 (0.12–0.81)^+^	0.45 (0.24–0.81)*	0.21 (0.12–0.38)*	0.27 (0.16–0.44)*	0.53 (0.20–1.41)
Income (Ref: < $50,000)						
≥ $50,000	0.61 (0.43–0.86)*	1.65 (0.62–4.37)	0.75 (0.41–1.37)	0.79 (0.45–1.41)	0.60 (0.33–1.09)	0.79 (0.20–3.06)
Missing	1.26 (0.66–2.42)	0.56 (0.13–2.42)	0.45 (0.19–1.09)	0.91 (0.36–2.33)	0.64 (0.33–1.27)	0.15 (0.03–0.83)^+^
Housing status (Ref: own)						
Other	0.61 (0.43–0.86)	1.89 (0.76–4.71)	1.29 (0.65–2.57)	2.37 (1.46–3.82)*	0.76 (0.46–1.27)	0.80 (0.27–2.44)
Residential area, Oahu	1.26 (0.66–2.42)	0.77 (0.31–1.95)	0.80 (0.46–1.40)	1.30 (0.84–2.02)	1.16 (0.76–1.76)	0.93 (0.45–1.92)
Have healthcare coverage	0.61 (0.43–0.86)^+^	2.72 (0.60–12.31)	2.19 (0.80–5.99)	1.51 (0.62–3.68)	1.35 (0.70–2.59)	3.13 (0.72–13.54)
Couldn’t see doctor because of medical cost	1.26 (0.66–2.42)*	1.42 (0.19–10.79)	1.56 (0.83–2.93)	1.20 (0.58–2.50)	2.15 (1.32–3.53)*	1.77 (0.70–4.50)
*Need variable*						
Exercise	0.39 (0.28–0.53)*	0.60 (0.25–1.47)	0.61 (0.35–1.07)	0.28 (0.17–0.46)*	0.38 (0.25–0.58)*	0.38 (0.17–0.82)^+^
Heavy drinking	0.64 (0.42–0.97)^+^	0.86 (0.11–6.97)	0.19 (0.03–1.02)	1.23 (0.50–3.04)	1.69 (0.88–3.24)	1.64 (0.53–5.08)
Smoking (Ref: current)						
Former/never	0.91 (0.64–1.29)	0.58 (0.22–1.55)	1.06 (0.47–2.42)	1.05 (0.48–2.30)	0.67 (0.44–1.02)	1.66 (0.75–3.65)
H/O heart attack	1.74 (1.17–2.60)*	0.34 (0.03–4.34)	1.65 (0.38–7.08)	0.42 (0.14–1.22)	1.73 (0.93–3.21)	7.57 (1.8–31.88)*
H/O CHD	1.26 (0.78–2.05)	6.02 (0.90–40.3)	0.96 (0.28–3.32)	4.71 (2.19–10.14)*	0.87 (0.43–1.77)	1.40 (0.28–7.01)
H/O stroke	2.07 (1.33–3.24)*	1.11 (0.17–7.12)	3.21 (1.16–8.84)^+^	0.80 (0.37–1.74)	1.71 (0.83–3.52)	2.35 (0.46–11.9)
H/O asthma	1.46 (1.06–2.02)^+^	1.78 (0.67–4.70)	1.29 (0.63–2.62)	0.65 (0.35–1.21)	1.44 (0.91–2.28)	1.94 (0.84–4.50)
H/O cancer	1.12 (0.84–1.48)	1.07 (0.35–3.28)	1.90 (0.64–5.59)	1.44 (0.80–2.59)	1.28 (0.64–2.57)	1.89 (0.76–4.73)
H/O COPD	1.65 (1.10–2.47)^+^	1.47 (0.30–7.16)	1.46 (0.50–4.22)	1.35 (0.53–3.45)	2.63 (1.23–5.65)^+^	1.17 (0.43–3.18)
H/O arthritis	2.06 (1.54–2.74)*	2.09 (0.84–5.20)	3.36 (1.74–6.50)*	2.83 (1.70–4.72)*	1.21 (0.74–1.97)	1.98 (0.82–4.78)
H/O depression	6.12 (4.60–8.16)*	5.82 (1.90–17.87)*	18.04 (9.30–35.01)*	7.00 (4.47–10.97)*	5.23 (3.47–7.88)*	15.67 (6.95–35.37)*
H/O kidney disease	1.22 (0.70–2.12)	1.29 (0.35–4.75)	1.66 (0.70–3.92)	1.60 (0.73–3.48)	1.77 (0.94–3.34)	0.92 (0.34–2.50)
H/O diabetes	0.84 (0.58–1.21)	2.06 (0.86–4.93)	1.40 (0.75–2.60)	0.89 (0.45–1.76)	1.42 (0.85–2.36)	1.42 (0.49–4.07)
Obese/overweight	0.91 (0.64–1.29)	0.43 (0.16–1.14)	0.60 (0.34–1.06)	1.00 (0.62–1.61)	0.94 (0.57–1.54)	1.34 (0.59–3.02)

Activity limitation was dichotomized as ≥ 14 unhealthy days (1) vs. < 14 unhealthy days (0). A multivariable logistic regression with quasi-binomial distribution was conducted, accounting for the complex sampling design

NHPI, Native Hawaiian/other Pacific Islander; H/O, history of; CHD, coronary heart disease; COPD, chronic obstructive pulmonary disease; Ref, reference

^+^*P* value < 0.05; *P value < 0.01

Predictors specific to White were one from predisposing variables, three from enabling variables, and four from need variables: age, employment (OR = 0.30), income, medical cost affordability, exercise (OR = 0.39), heart attack (OR = 1.74), stroke (OR = 2.07), and arthritis (OR = 2.06). People aged 65 years or older are less likely to be distressed compared to those aged 18–44 years (OR = 0.44). Income was associated with distress in activity limitation only for White and those having ≥ $50,000 were less likely to be distressed compared to those having < $50,000 (OR = 0.61). Those who couldn’t see a doctor due to medical cost were 1.26 times more likely to be distressed than those who can.

For Chinese, the common variable, depression, was only significantly associated with distress in activity limitation. People with depression was 5.82 times more likely to be distressed than those without.

Predictors specific to Filipino were one from enabling variables and one from need variables: employment (OR = 0.45) and arthritis (OR = 3.36). In addition, although stroke was not significant at 0.01 (P = 0.024), its effect was the highest among all racial/ethnic groups (OR = 3.21).

Specific to Japanese, two from enabling variables and three from need variables were significant: employment (OR = 0.21), housing status, exercise (OR = 0.28), CHD (OR = 4.71) and arthritis (OR = 2.83). Housing status was significant only for Japanese. People who rent or live in other arrangements were 2.37 times more likely to be distressed than those who own their home.

Specific to NHPI, one from predisposing variables, two from need variables, and one from need variables were significantly associated with distress in activity limitation: age, employment (OR = 0.27), medical cost affordability and exercise (OR = 0.38). Those aged 65 years or older are less likely to be distressed compared to those aged 18–44 years (OR = 0.20). People who couldn’t see a doctor due to medical cost were 2.15 times more likely to be distressed than those who can. Although COPD was not significant at 0.01 (*P* = 0.013), its effect was the highest among the racial/ethnic groups (OR = 2.63).

A predictor specific to Hispanic was heart attack from need variables. People who reported they had a heart attack were 7.57 times more likely to report distress in activity limitation than their counterparts.

#### HRQOL by race/ethnicity

We summarized the variables that were significantly associated with at least one of the HRQOL measures for each racial/ethnic group (see “Appendix”). For White, eight variables appeared to be significant to at least three HRQOL measures. The variables were age from predisposing variables, employment and medical cost affordability from enabling variables; and exercise, heart attack, stroke, arthritis, and depression from need variables. For Chinese, only depression was significant to at least three HRQOL measures. For Filipino, two variables from need variables were significant to at least three HRQOL measures: arthritis and depression. For Japanese, exercise, arthritis, and depression were significantly associated with at least three measures. For NHPI, four need variables of exercise, COPD, arthritis, and depression appeared to be significant for at least three HRQOL measures. For Hispanic, only depression was significantly associated with at least three measures.

## Discussion

In this study, we found significant racial/ethnic differences in the four HRQOL measures and identified variables associated with each of the HRQOL measures by race/ethnicity. To the best of our knowledge, this is the first study that disaggregated Asian groups to recognize association with different HRQOL measures by the distinct racial/ethnic groups.

### Racial/ethnic difference in HRQOL measures

Race/ethnicity was associated with all four HRQOL measures, but the directions and effects differed across the measures. We found all minority ethnic groups have higher distress rates in general health than White, consistent with the findings from other studies [17, 18]. However, the directions of the other three HRQOL measures among Filipino and Japanese were opposite to that of general health; these ethnic groups displayed significantly lower distress rates than White. This is not a novel finding for Japanese. A study conducted using California Health Interview Survey data reported that elderly Japanese Americans were healthier in many indicators related to health status and health behavior than other ethnic groups in California [19]. The reason for these conflicting trends between general health and the other HRQOL measures among Japanese are unclear; but this may be related to their culture, genetics, and living styles such as diet. Coe and his colleagues revealed that Japanese Americans have healthier profiles in inflammatory biology associated with aging and chronic condition than White and Black [20].

On the other hand, we did not expect the lower distress rates in the three HRQOL measures among Filipinos, because worse health outcomes have been reported for Filipino Americans [21, 22]. We presume that different distributions in the characteristics among Filipinos in Hawaii might be the reason. Filipinos in Hawaii were generally younger and have fewer chronic conditions than White. Consistent with our finding, other studies also reported a lower rate of physical health among Filipinos compared to Whites [23, 24]. Future study will be needed to validate our finding and to compare Filipino’s perceived HRQOL measures among different states.

Our study showed a lower distress rate in activity limitation in Chinese ethnic group compared to White. Studies on activity limitation including Chinese are lacking. To our knowledge, only a few studies have investigated HRQOL with this ethnic group. Gee and Ponce explored associations between racial discrimination, limited English proficiency, and HRQOL with Asian ethnic groups in California and reported the lowest distress rate in activity limitation in Chinese among Asian ethnic groups [13].

Different from the Asian ethnic groups, NHPI and Hispanic ethnic groups revealed higher distress rates in HRQOL measures compared to White. Compared to White, distress rates were significantly higher in all four measures among NHPIs and only in mental health among Hispanic. These ethnic groups have consistently reported worse physical and/or mental health than other racial/ethnic groups and known to experience various health-related issues including substance abuse, depression, and obesity [25–28].

### Variables associated with HRQOL measures by race/ethnicity

In this study we identified different factors for HRQOL for the six major racial/ethnic groups in Hawaii. None consistently associated with all the four HRQOL measures across all racial/ethnic groups. All racial/ethnic groups had at least one item related to need variables that predicted HRQOL measures.

For the majority of the racial/ethnic groups, our study suggests that HRQOL may be improved by increasing physical activity and preventing depression. Similar to other studies [29, 30], exercise was strongly associated with at least one of the HRQOL measures except for Filipinos. Our finding highlights the importance of developing physical activity programs and of encouraging people to engage in physical activity, which will help reduce adverse health outcomes and improve HRQOL. For history of depression, the significant association with mental health as well as the other HRQL measure implies that depression management plan can help enhance HRQOL. O’Neil et al. demonstrated that depression treatment significantly improved mental health in a meta-analysis on randomized controlled trials for cardiac patients [31]. In our study, the effects of depression were extremely high for Hispanic group on mental health and activity limitation (OR > 9.0). Some studies found that migration and longer residency for Hispanic in the U.S. were associated with progressively higher rates of internalizing mental disorders [32, 33]. This ethnic group may experience stressors attributed to migration such as acculturation stress and limited English proficiency. Therefore, further research is required to investigate the association between HRQOL and immigration including this ethnic group. As noted by Gaynes and his colleagues [34], since depression and chronic conditions interact to increase their effects, adopting a multidimensional approach would be ideal rather than treating depression or chronic condition alone.

In addition, we revealed that history of arthritis is an important factor for at least two of the HRQOL measures for all the racial/ethnic groups except for Hispanic, consistent with a previous study [35]. Patients with arthritis are more likely to report persistent pain, functional disability, fatigue, and depression, despite arthritis-related treatments [36]. Bolen et al. reported a lower prevalence rate of arthritis but a higher rate of arthritis-attributable activity limitation among Hispanics compared to Whites [37]. Our data also showed Hispanic had a lower rate in history of arthritis. Though it may be due to relatively small sample size for this ethnic group in our study, it is still unclear why arthritis was not a risk factor among Hispanic for HRQOL, which merits further investigation.

Not many predisposing variables appeared to be important. Age was significant for at least one of the HRQOL measures except for Chinese. Regardless of its significance or race/ethnicity, our finding underscores different directions between general health and mental health among most racial/ethnic groups. The rate of distress in general health shows a curve shape by age. People in the age group of 45–64 years have highest rate. Many people in this age group are approximately in menopause (or andropause for male). Because of hormone changes, the prevalence rate of chronic conditions and obesity increases so people in menopause feel and see their health differs from what it used-to-be. The distress rate in general health decreased after this age group, in which resilience may play an important role with age. Resilience refers to “successful adaptation and swift recovery after experiencing life adversities” [38]. Older adults might adapt their view on their physical health to be more positive as they adjust to life with physical changes and chronic illness. In addition, our study revealed people aged 65 years or older had the smallest rate of having mentally unhealthy days. Consistent with findings from other studies [39, 40], this might indicate that older adults appear to be mentally more stable. According to Blanchard and Coats [41], older adults reported fewer problematic and ambivalent relationship and fewer interpersonal stressors such as arguments and disagreements than younger adults. However, activity limitation was significantly lower in the age group aged 65 years or more compared to the age group of 18–44 years. Since the question for activity limitation is related to both physical and mental health, the significant mental health seems to be the main contributor to the lowest activity limitation in this age group.

Employment and medical cost affordability were associated with at least one of the HRQOL measures except for Hispanic and Hispanic/Filipino, respectively. This indicates that HRQOL can be improved by increasing support for medical expenditure for some of the economically disadvantaged. As Chaffee et al. suggested [11], there is a need for proportionate (targeting the disadvantaged) and universal (assessable to all) action plans in the U.S. to enhance equity and direct preventive efforts, through concerted support of the disadvantaged.

### Clinical implications

Our study provides some insight with clinical and public health implications in Hawaii, a state with diverse racial/ethnic subpopulations. Racial/ethnic differences emphasize the importance of tailoring interventions for specific racial/ethnic groups to improve individual’s health outcomes and quality of life. Although some progress has been made toward elimination of racial/ethnic health disparities, significant disparities persist, particularly for NHPI [42, 43]. This ethnic group has suffered disproportionate burdens of many chronic health conditions compared to White. Race/ethnicity is associated with many modifiable enabling and need variables and the culture of race/ethnicity can affect a person’s behavior, coping style and healthcare decision-making [44]. Although race/ethnicity is not modifiable, incorporating the cultures of the disadvantaged racial/ethnic groups into interventions may help reduce health disparities, especially for the variables specifically associated with those racial/ethnic groups. Some intervention studies found that mHealth (e.g., youtube, text message) can deliver mental health knowledge or improve mental health among Chinese Americans [45, 46]. Although direct interventions to improve HRQOL may not be possible, indirect interventions to change characteristics associated with HRQOL could result in improvement.

### Limitations and strengths

We want to point out several limitations in interpreting the study results. First, we could not account for other potential predictors (e.g., activities of daily living, adverse childhood experiences [47], and visual impairment [48]) that were not available in the Hawaii BRFSS database. Second, the data are cross-sectional so we could not explore the temporal change of HRQOL or causal effect between healthcare utilization and HRQOL. Third, because of the small subgroup sizes, some races such as other Asians, American Indians, and Blacks were combined to obtain more robust results. Fourth, the HRQOL measures are self-reported so they might be mis-classified and subject to response bias and measurement errors. Fifth, there may be some hidden or latent structures of the variables used in the study. The four HRQOL measures were moderately correlated (correlation coefficients ranging from 0.224 to 0.587), but they were associated with different variables in various directions and magnitudes. We did not conduct more advanced analysis such as a structural equation model, in the current study. However, this brings up a need for highlighting the need for multidimensional constructs of variables including HRQOL.

Despite these limitations, our study has several strengths. The use of a representative dataset makes it possible to generalize our findings of HRQOL perceptions to the Hawaii adult population. The use of the detailed racial information allows us to identify differences in the HRQOL among NHPI and various Asian ethnic groups. Finally, investigating the four HRQOL measures together is an additional strength of the current study.

## Conclusion

This study contributes to the literature on disparities in HRQOL and predictors of HRQOL among diverse racial/ethnic subgroups. The factors associated with HRQOL by different racial/ethnic groups will provide valuable information for identifying future public health priorities to improve quality of life, and will help state governments and healthcare professionals remove the potential barriers to increase healthcare utilization.

## Data Availability

The data used for this study can be obtained from the Hawaii State’s Department of Health.

## Appendix: Important variables for health-related quality of life by race/ethnicity

**Table:**

Variable	White	Chinese	Filipino	Japanese	NHPI	Hispanic
Predisposing	*Age*^a,c,d^; Education^a,c^		Age^b^	Age^a,b^; Education ^a^	Sex^b^; Age^c,d^; Marital status^b^	Age ^a^
Enabling	*Employment*^a,b,d^; Income^a,d^; Affordability of medical cost^a,b,c,d^	Employment^b^; Affordability of medical cost^c^	Employment^d^	Employment^b,d^; Income^a^; Affordability of medical cost^a,c^; Residential area^b,d^	Employment^d^; Affordability of medical cost^c,d^	Residential area^a^
Need	Exercise^a,b,c,d^; Smoking^c^; *Heart attack*^a,c,d^; *Stroke*^a,b,d^; Cancer^a,b^; COPD^a,b^; *Arthritis*^a,b,d^; Depression^a,b,c,d^; Kidney disease^a^; Diabetes^a^; Obese/overweight^a^	Exercise^a,b^; Asthma^b^; Cancer^a^; COPD^a,b^; Arthritis^a,b^; *Depression*^a,c,d^; Kidney disease^a,b^; Diabetes^a^	Smoking^a^; Stroke^b^; CHD^a^; Cancer^a^; *Arthritis*^a,b,d^; Depression^a,b,c,d^; Kidney disease^a,b^; Diabetes^a^	*Exercise*^a,b,d^; Heavy drinking^c^; CHD^a,d^; Cancer^a,b^; COPD^a,b^; Arthritis^a,b,c,d^; Depression^a,b,c,d^; Kidney disease^a^; Diabetes^a,b^; Obese/overweight^a^	*Exercise*^a,b,d^; Smoking^a^; *COPD*^a,b,c^; *Arthritis*^a,b,c^; Depression^a,b,c,d^; Kidney disease^a^; Diabetes^a^; Obese/overweight^a^	Exercise^a^; Heart attack^d^; Asthma^c^; *Depression*^b,c,d^; Diabetes^a^

Health-related quality of life (HRQOL) was assessed by four measures: general health, physical health, mental health, and activity limitation. General health was dichotomized as fair/poor (1) vs. excellent/very good/good (0). Physical health, mental health, and activity limitation was dichotomized as ≥ 14 unhealthy days (1) vs. < 14 unhealthy days (0).

NHPI, Native Hawaiian/other Pacific Islander; CHD, coronary heart disease; COPD, chronic obstructive pulmonary disease.

A multivariable logistic regression with quasi-binomial distribution was conducted for each of HRQOL measures, accounting for the complex sampling design at a significance level of 0.01: ^a^Significant factor for general health. ^b^Significant factor for physical health. ^c^Significant factor for mental health. ^d^Significant factor for activity limitation.

The variables in a column are the ones significantly associated with at least one of four HRQOL measures for the corresponding racial/ethnic group. A variable in underline indicates that it was significantly associated with all four HRQOL measures; A variable in italic indicates that it was significantly associated with three of the HRQOL measures.

## Ethnical approval

The Institutional Review Boards (IRBs) of University of Hawaii approved this study as exempt (#2017-00821).
